# Clinico-demographic Profile of Patients Presenting with Road Traffic Accidents at National Trauma Center of Nepal: An Observational Study

**DOI:** 10.31729/jnma.8843

**Published:** 2024-12-31

**Authors:** Badri Rijal, Bikash Kc, Amartya Dahal, Nabaraj Gautam, Min Chandra Adhikari

**Affiliations:** 1 Department of Orthopedics, National Trauma Center, Mahankhal, Kathmandu, Nepal; 2 Kathmandu University School of Medical Sciences, Dhulikhel Hospital, Dhulikhel, Kavre, Nepal; 3 National Trauma Center, Mahankhal, Kathmandu, Nepal; 4 Department of Internal Medicine, National Trauma Center, Mahankhal, Kathmandu, Nepal

**Keywords:** *accidents*, *injuries*, *road traffic accident*, *trauma*

## Abstract

**Introduction:**

Road traffic accidents are a major global health concern, accounting for 1.35 million fatalities and countless impairments every year. The number of road traffic accidents in Nepal is rising, which has a significant effect on the country's economy and healthcare system. Over a period of five years, the study aimed to analyze the clinico-demographic characteristics of patients admitted for injuries due to road traffic accidents in the National Trauma Center of Nepal.

**Methods:**

This is an observational study conducted by reviewing a five-year admission data from 2018 to 2023 A.D. of a tertiary care trauma center. The study was conducted following approval from the Ethical Review Board of Nepal Health Research Council (Reference number: 969). All patients admitted to the trauma centre following injuries due to road traffic accidents were included in the study. The results were summarised using descriptive statistics.

**Results:**

Out of 20,843 admissions, 3,810 (18.28%) were due to RTAs. The male-to-female ratio was 4.44:1, with a median age of 31.00 (24.00 to 43.00) years. Age distribution was as follows: <18 years (6.75%), 18-39 years (62.13%), 40-59 years (23.62%), and ≥60 years (7.50%). The median hospital stay was 8.00 (3 to 16) days. The most common injuries were lower limb 2386 (62.62%), upper limb 958 (25.14%), and head injuries 890 (23.36%). The mortality rate was 72 (1.89%).

**Conclusions:**

Injuries due to road traffic accidents constitute a significant portion of admissions at the National Trauma Center in Nepal, predominantly affecting young males. Lower limb injuries were most common.

## INTRODUCTION

Globally, traffic accidents pose significant health challenges and are a focal point for healthcare institutions. Annually, approximately 1.35 million individuals either face death or endure disabilities due to these incidents.^1^ According to the World Health Organization (WHO), the economic toll of road traffic crashes typically amounts to approximately 3% of a country's Gross Domestic Product (GDP).^2^

Trauma is a leading cause of death in low and middle-income countries (LMICs), where preventive measures and healthcare systems remain underdeveloped.^3^ In Nepal, the burden of injuries is rising, with deaths from injuries increasing from 6.31% to 9.21%.^4^ A hospital-based injury surveillance study identified road traffic accidents (RTAs) as the most frequent injuries presented in emergency departments.^5^ Moreover, the mortality rate due to RTAs increased from 4 per 100,000 in 2001-2002 to 7 per 100,000 in 2011-2012.^6^

The study aimed to analyze the clinical demographic profile of patients presenting due to Road Traffic Accident (RTA) admitted to the National Trauma Center.

## METHODS

This observational cross-section study was conducted at National Trauma Centre, which is a tertiary care trauma center of Nepal. A five-year retrospective data from January 1, 2018 to December 31, 2023 (2075 to 2080 B.S.) was analyzed after approval from the Ethical Review Board of Nepal Health Research Council (Reference number: 969). Data was retrieved from the record section and checked for completeness. Records of all patients admitted to the hospital within the study period were included in the study. Those patients with duplicate records or incomplete records were excluded. Total sampling was done.

Demographic variables, patterns of injuries, site of injuries, Data was collected with the help of a proforma and recorded in Microsoft Excel. The data was checked for completeness and analyzed using IBM SPSS Statistics for Windows, version 25.6 (IBM Corp., Armonk, N.Y., USA). Descriptive statistics (mean, median, frequency, standard deviation, and percentage) were applied.

## RESULTS

There were a total of 20,843 cases admitted to the National Trauma Center in five years. Among them, 3810 (18.28%) admissions were due to road traffic accidents. The male-to-female ratio was 4.44:1 with 3109 (81.60%) male and 700 (18.40%) female. The mean age of the patients was 34.76±14.57. The distribution of patients by age category is as follows: Less than 18 years 257 (6.75%), 18-39 years 2367 (62.13%), 4059 years 900 (23.62%), and 60 years and above 286 (7.50%). The median hospital stay was 8 (3 to 16) days.

During this study period, there were 2386 (62.62%) lower limb injuries, 958 (25.14%) upper limb injuries, 890 (23.36%) head injuries, 285 (7.48%) vertebral/spine injuries. There were 124 (5.17%) thoracic injuries and 163 (4.28%) abdominal injuries (Table 1).

**Table 1:** Types injuries in patients admitted due to road traffic accident (n=3810).

Type of injury	n (%)
**Head Injuries (n=890)**	
EDH	121 (13.60)
SDH	107 (12.02)
SAH	82 (9.21)
Intracerebral Hemorrhage	21 (2.36)
Intraventricular hemorrhage	14 (1.57)
Others intracranial hemorrhages	2 (0.22)
Cranial bone fracture	152 17.08)
Facial bone fracture	268 (30.11)
Pneumocephalous	92 (10.33)
Miscellaneous Head Injuries	31 (3.48)
**Vertebral/Spine Injuries (n=285)**	
Fracture of First Cervical Vertebra	12 (4.21)
Fracture of Second Cervical Vertebra	25 (8.77)
Fracture of other cervical vertebra	29 (10.18)
Fracture of Thoracic Vertebra	90 (31.58)
Fracture of Lumbar Vertebra	68 (23.86)
Fracture of Sacrum vertebrae	3 (1.05)
Vertebral Translation and Subluxation	37 (12.98)
Spinal cord injury	20 (7.02)
Miscillenous vertebral/spine injury	1 (0.35)
**Upper Limb Injuries (n=958)**	
Shoulder Joint Dislocation	26 (2.71)
Radius fracture	233 (24.32)
Humerus fracture	192 (20.04)
Clavicular fracture	174 (18.16)
Ulnar fracture	126 (13.15)
Both radius and ulnar fracture	45 (4.70)
Miscelleneous fracture	99 (10.33)
Brachial plexus injury	47 (4.90)
Miscellaneous upper limb injuries	16 (1.67)
**Lower Limb Injuries (n=2386)**	
Fracture of pelvis	146 (6.12)
Dislocation of hip	28 (1.17)
Fracture of femur	522 (21.88)
Fracture of patella	71 (2.98)
Injury to ligament of knee	122 (5.11)
Dislocation of knee	13 (0.54)
Fracture of tibia	809 (33.91)
Fracture of fibula	316 (13.24)
Fracture of both tibia and fibula	192 (8.05)
Ankle dislocation/subluxation	2 (0.08)
Fracture of tarsal bone	35 (1.47)
Fracture of metatarsal	38 (1.59)
Miscellaneous	92 (3.86)
**Thoracic Injuries (n=124)**	
Single rib fraacture	10 (8.06)
Multiple rib fracture	63 (50.80)
Abnormalities of pleural space (hemothorax, pneumothorax, hemopneumothorax)	43 (34.68)
Others	8 (6.45)
**Abdominal injuries (n=163)**	
Hemoperitoneum	49 (30.06)
Livery injury	38 (23.31)
Spleen injury	34 (20.86)
Hollow viscus perforation	19 (11.66)
Miscellaneous abdominal injury	23 (14.11)

SDH=Sub Dural Hemorrhage, EDH=Extra Dural Hemorrhage, SAH = Sub Arachnoid Hemorrhage

Out of all patients who were admitted 3504 (91.97%) patients recovered, 203 (5.33%) left against medical advice or discharged or absconded, 31 (0.81%) were referred to another centre, and there were 72 (1.89%) mortality.

## DISCUSSION

In this study, 3810 (18.28%) of patients were admitted due to road traffic accidents which is higher than a similar hospital-based cross-sectional study.^7^ Since our study site is a referral center for cases of trauma around Nepal, the prevalence of patients admitted due to road traffic accidents may be higher. In a study conducted by Karkee et al., secondary data analysis of traffic police records and systematic literature review which mostly included cross-sectional studies revealed that the predominant demographic affected by RTIs comprised males in the age range of 20 to 40 years.^6^ Our study showed similar results where 2376 (62.1%) of admitted patients were in the age group of 18-39 years and there was male predominance with male to female ratio of 4.44:1 The result was also similar to other hospital-based studies.^7,8^

In this study, lower limb injuries 2386 (62.62%) were the most common among admitted patients followed by upper limb injuries 958 (25.14%) and head injuries 890 (23.36%). Among lower limb injuries, tibia/fibula fracture was the most common 55.2% of lower limb injuries seen in our study, which is similar to a hospital-based cross-sectional study conducted by Mengistu.^9^ Femoral fracture has also been reported as the most common extremity fracture in another study, which is not the case in our study comprising 22.9% of lower limb injury cases.^10^ In a study conducted by Kushwaha et al., lower limb injury was the most common injury pattern, which was similar to our study,^11^ however In a study conducted by Pradhan et al., head injury and soft tissue injury were the most common injury patterns seen in patients presenting to the emergency department of a tertiary care center in Nepal.^7^

In our study, 890 (23.36%) of the population had head injury which is fewer in comparison to other hospital-based cross-sectional studies.^7,12^ Head injuries in our data set were characterized by predominant occurrences of intracranial hemorrhage types such as extradural 121 (13.6 %), subdural 107 (12.02 %), and subarachnoid hemorrhages 82 (9.21 %). A total of 420 (47.19 %) cases presented with skull fracture, which included various cranial and facial fracture. Extradural hemorrhage was the most common injury pattern followed by subdural hemorrhage in our study similar to a study conducted by Devkota et al.^13^ In patients with fatal head injuries, study from autopsy studies revealed subdural hemorrhage was the most common intracranial bleed.^14^

Upper limb injuries predominantly involved fractures, particularly radius fractures 233 (24.32%), as the most common. Thoracic trauma mainly featured rib fractures 73 (58.86%), while abdominal trauma frequently resulted in hemoperitoneum 49 (30.06%). Vertebral injuries included fractures of the thoracic 90 (31.58%).

The observed patterns of traumatic injuries can be attributed to various factors, such as the mechanism of injury, the energy involved, and the specific anatomical characteristics of the affected regions. For instance, a study on the patterns of bone fractures in relation to trauma energy found that high-energy injuries, such as those sustained in motor vehicle accidents, were more likely to result in complex fractures involving multiple bones or joints, whereas low-energy injuries, such as simple falls, were more commonly associated with isolated fractures.^15^

The mortality rate in our study was 72 (1.89%) among patients admitted due to road traffic accidents in our center. The mortality was less as compared to similar hospital-based cross-sectional studies.^7,12^ This study doesn't account for the cases of mortality from emergencies and those who were brought dead which may account for lower mortality in our case.

The study relies on retrospective data from hospital records, which may contain inaccuracies or incomplete information. This can introduce bias and limit the reliability of the findings. The cross-sectional design of the study does not allow for the examination of long-term outcomes or trends over time. This limits the ability to assess the causal relationships between variables. Certain potential confounding factors, such as the severity of injuries, pre-existing health conditions, etiology, and the circumstances of the accidents, were not properly recorded in the hospital data, limiting the depth of the analysis. There may be inconsistencies in how different healthcare providers record and report data, introducing reporting bias leading to variations that could affect the study's findings. Future multicenter and longitudinal studies are essential for a comprehensive understanding and effective intervention to mitigate the impact of RTAs in Nepal.

## CONCLUSIONS

The admission for trauma due to road traffic accidents were more than those reported in studies of similar setting. The admission population included predominantly males aged 18-39. Lower limb injuries were most common, followed by upper limb and head injuries, including intracranial hemorrhages and fractures.
